# Crowdsourcing for Machine Learning in Public Health Surveillance: Lessons Learned From Amazon Mechanical Turk

**DOI:** 10.2196/28749

**Published:** 2022-01-18

**Authors:** Zahra Shakeri Hossein Abad, Gregory P Butler, Wendy Thompson, Joon Lee

**Affiliations:** 1 Department of Biomedical Informatics Harvard Medical School Harvard University Boston, MA United States; 2 Data Intelligence for Health Lab Cumming School of Medicine University of Calgary Calgary, AB Canada; 3 Centre for Surveillance and Applied Research Public Health Agency of Canada Ottawa, ON Canada; 4 Department of Community Health Sciences Cumming School of Medicine University of Calgary Calgary, AB Canada; 5 Department of Cardiac Sciences Cumming School of Medicine University of Calgary Calgary, AB Canada

**Keywords:** crowdsourcing, machine learning, digital public health surveillance, public health database, social media analysis

## Abstract

**Background:**

Crowdsourcing services, such as Amazon Mechanical Turk (AMT), allow researchers to use the collective intelligence of a wide range of web users for labor-intensive tasks. As the manual verification of the quality of the collected results is difficult because of the large volume of data and the quick turnaround time of the process, many questions remain to be explored regarding the reliability of these resources for developing digital public health systems.

**Objective:**

This study aims to explore and evaluate the application of crowdsourcing, generally, and AMT, specifically, for developing digital public health surveillance systems.

**Methods:**

We collected 296,166 crowd-generated labels for 98,722 tweets, labeled by 610 AMT workers, to develop machine learning (ML) models for detecting behaviors related to physical activity, sedentary behavior, and sleep quality among Twitter users. To infer the ground truth labels and explore the quality of these labels, we studied 4 statistical consensus methods that are agnostic of task features and only focus on worker labeling behavior. Moreover, to model the meta-information associated with each labeling task and leverage the potential of context-sensitive data in the truth inference process, we developed 7 ML models, including traditional classifiers (offline and active), a deep learning–based classification model, and a hybrid convolutional neural network model.

**Results:**

Although most crowdsourcing-based studies in public health have often equated majority vote with quality, the results of our study using a truth set of 9000 manually labeled tweets showed that consensus-based inference models mask underlying uncertainty in data and overlook the importance of task meta-information. Our evaluations across 3 physical activity, sedentary behavior, and sleep quality data sets showed that truth inference is a context-sensitive process, and none of the methods studied in this paper were consistently superior to others in predicting the truth label. We also found that the performance of the ML models trained on crowd-labeled data was sensitive to the quality of these labels, and poor-quality labels led to incorrect assessment of these models. Finally, we have provided a set of practical recommendations to improve the quality and reliability of crowdsourced data.

**Conclusions:**

Our findings indicate the importance of the quality of crowd-generated labels in developing ML models designed for decision-making purposes, such as public health surveillance decisions. A combination of inference models outlined and analyzed in this study could be used to quantitatively measure and improve the quality of crowd-generated labels for training ML models.

## Introduction

### Background

In recent years, social media data have been extensively used in different areas of public health [1-3], such as detecting outbreaks and emerging diseases [4,5], monitoring adverse drug reactions [6], and predicting or modeling health-related behaviors and outcomes [7-9]. Since 2011, Twitter has been the most popular form of social media used for public health communication [10,11]. In 2020, Twitter alone reported 500 million tweets generated per day from 145 million daily active users. A recent scoping review of 755 articles on digital public health surveillance shows that Twitter is the most studied of all platforms and the most used platform to study communicable diseases, behavioral risk factors, mental health, drug use, and vaccines [11]. In addition to the inherent limitations of social media data, such as lack of demographic data and biased populations, when integrated with complex data-driven models such as artificial neural networks, these publicly accessible resources can be used for population-level surveillance to complement traditional public health surveillance (eg, surveys) with faster and less costly longitudinal information.

Although linguistic annotation is crucial for developing machine learning (ML) and natural language processing (NLP) models, manual labeling of a large volume of data is a notorious problem because of its high cost and labor-intensive nature. In recent years, this problem has been tackled using crowdsourcing technologies such as Amazon Mechanical Turk (AMT) [12], Crowdflower [13], and Prolific Academic [13] to obtain relatively low-cost labeled data more quickly and easily. AMT is a software service operated by Amazon that allows users to crowdsource work, broken into microtasks called HITs (Human Intelligence Tasks), to a large number of workers who are compensated for each HIT completed. With the vast potential applications of crowdsourcing in public health [14-16], the research community has seen steady growth in the use of AMT in the past 10 years. The number of studies indexed in PubMed using the search term *Amazon Mechanical Turk* AND *public health* has increased sharply from 42 studies in 2015 to 118 studies in 2019.

However, because of the uncertain quality of AMT workers with unknown expertise, their labels are sometimes unreliable, forcing researchers and practitioners to collect information redundantly, which poses new challenges in the field. Given that in large-scale crowdsourcing tasks the same workers cannot label all the examples, measuring interannotator agreement and managing the quality of workers differ from those of a team of in-house expert workers. Despite the growing popularity of AMT for developing ML models in public health research, the reliability and validity of this service have not yet been investigated. At least several public health studies have used AMT for training data-driven ML models without external gold standard comparisons [17-21]. Ayers et al [17] used AMT to create a gold standard data set to develop predictive models to detect electronic nicotine delivery systems on social media. Yin et al [18] developed a scalable classifier to detect personal health mentions on Twitter based on a gold standard data set generated by AMT workers. The reliability of the crowd-labeled data set in this study was measured based on the agreement among workers.

Similarly, to characterize sleep quality using Twitter, McIver et al [19] used AMT for sentiment annotation of text data and used interannotator agreement to assess the reliability of workers. Reece et al [20] used AMT to build a data set and develop a prediction model to detect depression emergence and posttraumatic stress disorder in Twitter users. To control the quality of the data collected, they required the workers to have completed at least hundred tasks, with a minimum 95% approval rating. Although research has supported the efficacy of using reputation to evaluate the quality of crowdsourced data [22], the reliability of using this metric in developing ML-based digital public health systems has not yet been investigated. Thus, in this study, in addition to defining qualification requirements for AMT workers, we studied the reliability of crowd-generated training data for developing ML models in the context of public health surveillance. We used AMT to collect 296,166 labels for 98,722 unique tweets, labeled by 610 AMT workers, to develop ML models that can detect the physical activity, sedentary behavior, and sleep quality (PASS) of Twitter users.

### Objectives

The primary aim of this study is to evaluate the application of AMT for training data-driven ML models by analyzing the quality of crowd-generated labels. As the quality of crowd-generated labels, regardless of the type of the task being studied, is critical to the robustness of ML models trained based on these labels, we created a gold standard data set of labels and applied several statistical and ML-based models to assess the reliability of using the crowd-labeling task from different perspectives (eg, process, design, and inference). To interpret the results of our quality assessment and explore the effect of noisy labels on the applicability of inference models in dealing with these labels, our approach involved evaluating the performance of 4 consensus methods, which do not involve task features in their truth inference, and exploring their feasibility in improving the quality of crowd-labeled data. As these methods are modeled purely as a function of worker behaviors concerning labeling tasks, they cannot leverage the value of context-sensitive information (ie, the task’s meta-information) in their inference decisions. Thus, we collected additional features for our labeling data set and developed 7 ML models, including a deep learning (DL) model and a hybrid convolutional neural network (CNN) architecture to couple worker behaviors with the task’s meta-information when inferring the truth label. To detect and correct noisy labels, we also developed 5 pool-based active learners to iteratively detect the most informative samples (ie, samples with more uncertainty) and remove them from the validation set. Finally, we used SHAP (Shapley Additive Explanations) [23] to explore the contribution of different features, including worker behaviors and context-sensitive features, to the results of our supervised inference models.

## Methods

### Labels

**Figure 1:**
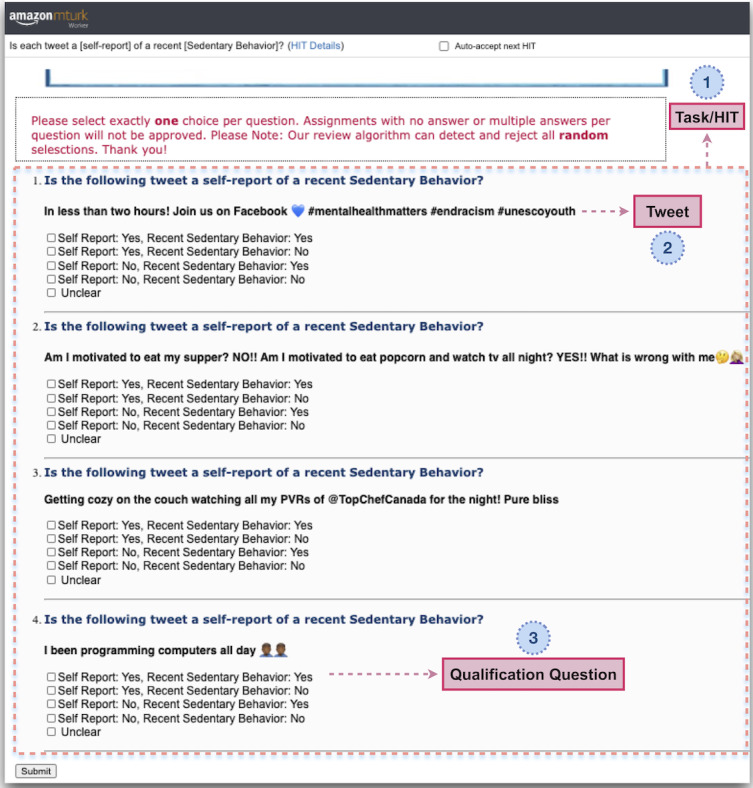
A sample labeling task (ie, human intelligence task [HIT]) for sedentary behavior. Each HIT contains 4 questions (section 1), and each asks if the presented tweet is a self-reported physical activity, sedentary behavior, or sleep quality–related behavior (section 2). The fourth question is an easy, qualification question that was used to check the quality of the worker (section 3).

The crowdsourcing tasks, referred to as HITs by AMT, were designed to collect 5 labels based on 2 conditions, self-reported and recent PASS experience, to develop binary and multiclass classification models that can detect PASS-related behavior in Twitter users. The labels of the multiclass prediction models were defined as 11, 10, 01, and 00, based on the value of each condition (Figure S1 in Multimedia Appendix 1). We also let workers choose a fifth option, called *unclear*, to ensure they did not give random labels to tasks they were not confident in performing successfully (Figure 1). We excluded this label for both inference and classification tasks. We defined the binary labels as 1 if both conditions were met and 0, otherwise. The binary labels did not directly come from the AMT workers and were generated by dichotomizing the collected labels.

### Crowdsourcing Workflow

We implemented a pipeline to create the HITs, post them on AMT, collect the labels through a quality check process, approve or reject the HITs, and store the results. To minimize noisy and low-quality data, we added a qualification requirement to our tasks and granted labeling access to workers who had demonstrated a high degree of success in performing a wide range of HITs across AMT (ie, master qualification). In addition, we added a simple qualification question to each HIT to detect spammers or irresponsible workers. Each HIT contained 4 questions, including the qualification question, and was assigned to 3 workers (Figure 1 and Figures S2 and S3 in Multimedia Appendix 1). Workers were asked to select exactly 1 choice per tweet, and HITs with zero or more than one label were rejected during the approval process. Through different iterations of data labeling, workers were paid from US $0.03 to US $0.05 after completing each HIT. We collected the labels for the 98,722 tweets used in this study through different iterations, from April 2019 to June 2020. We regularly checked the quality of the submitted tasks to detect low-quality workers during each iteration and revoke their access to our tasks. Before the formal initiation of the process, we pilot-tested the design, response time, and complexity of the HITs through 2 different iterations and revised the workflow accordingly. We did not collect any personally identifiable information from the workers (participants) during the data labeling task. The experiments were carried out in accordance with the relevant guidelines and the University of Calgary Conjoint Faculties Research Ethics Board regulations. We implemented the entire workflow in Python and used Boto3 Python software development kit to connect to and work with AMT.

### Data Collection

We collected data for this study from Twitter using the Twitter livestream application programming interface (API) for the period between November 28, 2018, and June 30, 2020. The data set was filtered to include only Canadian tweets relevant to PASS. A total of 103,911 tweets were selected from 22,729,110 Canadian tweets using keywords and regular expressions related to PASS categories. Each of these 103,911 tweets was labeled by 3 AMT workers, from which 98,722 tweets received 3 valid labels, with almost half of them related to physical activity.

The demographic variables of age and gender and the information about the source of each tweet (eg, organization vs real users) were not available within the data set collected from Twitter. We estimated these variables for each tweet using the M3 inference package in Python [24], which uses a multimodal deep neural architecture for the joint classification of age, gender, and information sources of social media data. The text (tweet) field and each of the daytime, weekday, and month variables were extracted from the metadata provided by the Twitter API.

We have made the Twitter data set used in this study publicly available [25].

### Data Processing

Tweets have a bounding box of coordinates, which enables spatial mapping to their respective city locations. As the Twitter API returns datetime values in Coordinated Universal Time, we used a time zone finder in Python and adjusted the time of each tweet based on its spatial data. Given that daytime, month, and weekday can be influential factors in twitting about each of the PASS categories, and to better use the datetime data (%a %b %d %H: %M: %S %Y), we extracted a: weekday, b: month, and H: hour fields and stored them as separate features.

We cleaned the text column by eliminating all special characters (eg, #, &, and @), punctuations, weblinks, and numbers. We also replaced common contractions with their uncontracted forms; for example, *I’ll* was resolved as *I will*. While developing and evaluating our NLP models, we noticed that the impact of removing stop words, stemming, and converting the text to lower case on the performance of our predictive models was not noticeable. This could relate to the ability of transfer-learning techniques (ie, GloVe embeddings) to generalize on unseen data. Thus, we applied neither stop-word removing nor lexical cleaning on the textual features of our data set. Moreover, as hashtags and emojis can be used as independent words and facilitate emotional expressions, we did not remove them during the cleaning process.

To develop the ML models, all categorical data were encoded into dummy variables using one-hot encoding, and as we only approved HITs with complete answers, this data set did not contain any missing data.

### Label Consistency

To measure the consistency of answers given by the workers, we calculated label consistency (LC) as the average entropy of the collected labels for each PASS category [26]. For each tweet *t_i_* ∈ *T_s_*, where *T_s_* denotes the set of all tweets related to surveillance category *s* ∈ {physical activity, sleep quality, sedentary behavior}, *n_ij_* defines the number of answers given to the *j^th^* choice (*j* ∈ {1,2,3,4,5}, as we had 5 choices for each tweet). We calculated *LC_s_* as follows:



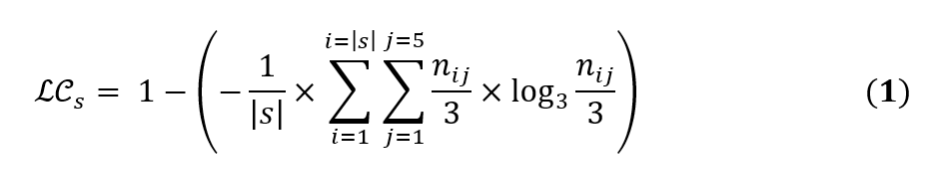



|s| denotes the size of the surveillance category *s* and, as we collected 3 labels for each tweet, the denominators in the entropy formula received a constant value of 3. *LC* ranges from 0 to 1, and values close to 1 show more consistency among the workers’ input.

### Ground Truth Data Set

To investigate the viability of unsupervised inference models in predicting truth labels from crowd-labeled data and compare it with that of supervised predictive models, we used a random sample of our data set as a ground truth set (ie, 9000 tweets: 4000 tweets for physical activity, 3000 tweets for sleep quality, and 2000 tweets for sedentary behavior). In total, 6 data scientists manually labeled this sample, and the entire labeled data set was reviewed manually and relabeled by an experienced in-house domain expert in both ML and public health surveillance. The disagreements between this data set and the crowd-labeled data set were manually checked to exclude any labeling bias that could impact the results of this study.

### Inference Models

The majority voting (MV) approach estimates the actual ground truth based on most labels submitted by different workers. For example, defining the estimated label as 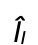, and the submitted label by worker *w* as *l_w_*, the MV approach, for a binary labeling task, assigns 1 to 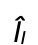 if 
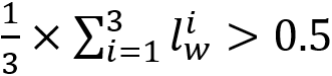
 and 0, otherwise. Although individual workers’ reliability coming from different backgrounds with different quality levels varies, the MV approach assumes equal expertise among the workers and does not model worker behaviors [27]. As this approach is completely task-independent, it does not involve task properties in the inference process; thus, it is fast.

The David and Skene (DS) [28] approach uses expectation–maximization (EM) to simultaneously estimate the error rate of annotators (workers) and latent label classes, when, similar to MV, the ground truth is unknown, and workers are assumed to operate independently. Unlike MV, which is agnostic to worker behavior, DS models worker k’s behavior as a function of each task’s true label by creating a confusion matrix *π^k^* with size *L* × *L*, where *L* is a fixed number and represents the number of possible labels for a single-labeled classification task. DS defines worker *k*’s error rate 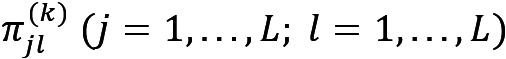 as follows:



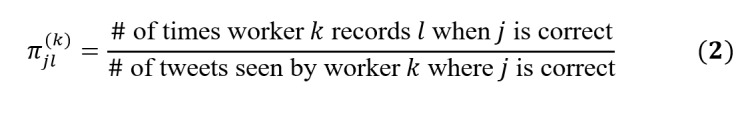



As not all workers need to label all the tasks, and a worker may label the same task more than once, sparsity can be a problem in large-scale labeling tasks when using the DS approach [27]. DS iteratively estimates the true label of each task based on the worker’s quality and estimates the worker’s error rate (quality) based on the inferred labels until it converges. Although the worker-specific confusion matrix generates the quality score of each worker, it may not be sufficient to measure the actual contribution of each worker [29]. The inherent complexity of a task, especially in NLP, or a worker’s bias may result in wrong labels, although the worker is quantitatively accurate.

The generative model of labels, abilities, and difficulties (GLAD) [30] models the quality of workers as a function of the input task using parameter *α*. The quality parameter ranges from –∞ to +∞, implying that the worker always labels the tasks incorrectly or correctly, respectively. When *α*=0, the worker cannot distinguish among the labels, and their input does not contribute to the task’s correct label. To estimate the ground truth, in addition to the workers’ quality, GLAD models the difficulty of task *t_i_* as *d_i_*=1/*β_i_*, where *β_i_*>0. The difficulty index ranges from 0 to ∞, where *d_i_*=∞ classifies *t_i_* as the most difficult task, and *d_i_*=0 means that the task always receives a correct label, even from the workers with *α*≤0. GLAD uses the EM approach to obtain the maximum likelihood estimation of *α* and *β*, and models the probability that worker *k* correctly labels *t_i_* using 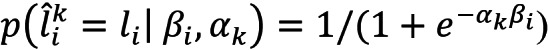.

Similar to DS, Raykar algorithm (RY) [31] forms a confusion matrix to model a worker’s quality. In addition, in the case of binary classification, it models worker’s bias toward the positive class (ie, sensitivity) and toward the negative class (ie, specificity) using beta prior [27]. Worker bias in this context usually occurs when a worker underestimates or overestimates the truth of a task [26]. As with DS and GLAD, RY uses an unsupervised EM approach to estimate each of the model parameters and truth labels. Depending on the availability of task-specific features, RY can either use automatic supervised classifiers or fall back to unsupervised EM models to estimate the truth label.

### Predictive Models

As the meta-information associated with each task may reveal its underlying complexity and thus help model worker behaviors, we developed a set of ML models to involve this metadata in the inference process. Models were trained based on quintuple *F*: (*W*,*I*,*M*,*t*,*l*), where *W* = {*w_1_*,...,*w_k_*} represents labels collected from AMT workers, *I* = {*MV*,*DS*,*RY*,*GLAD*} denotes the results of inference models, and *M* denotes metadata associated with each tweet including time (ie, weekday, month, and daytime), gender, age group, and the source of the tweet (ie, organization vs real people). The text of each tweet is presented by *t*, and *l* denotes the truth label.

**Figure 2:**
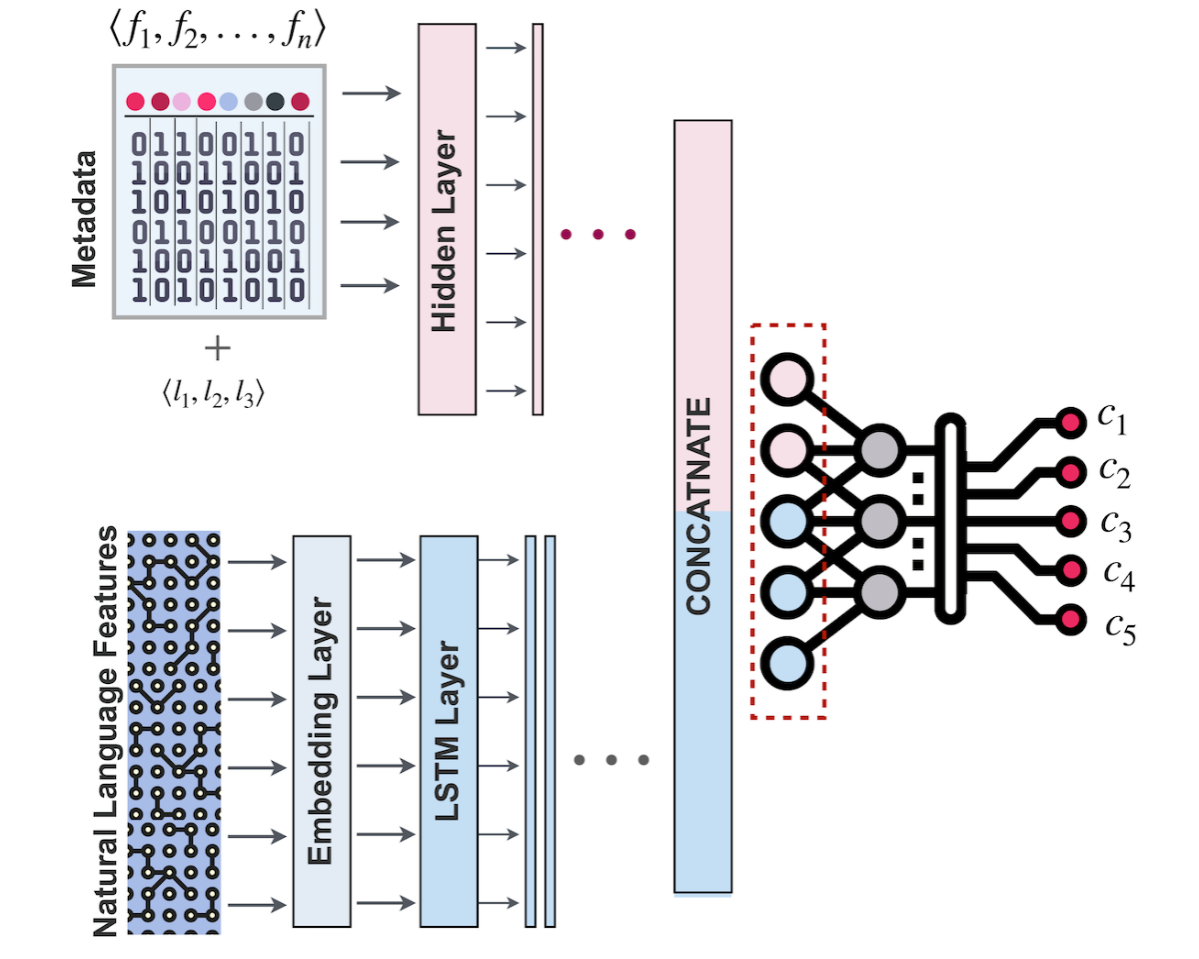
The pipeline of the deep learning model used to predict labels using both textual information and meta-information. LSTM: long short-term memory.

To mitigate the risk of biased results caused by a specific learning algorithm and overcome the overfitting problem, we developed and evaluated 5 standard ML classifiers with different architectures, including generalized linear (logistic regression [LR]), kernel-based (support vector machines [SVM]), decision-tree–based (random forest and XGBoost), and sample-based (K-nearest neighbors [KNN]) classifiers. Moreover, to incorporate textual features into our analysis, we developed a hybrid DL architecture in which a CNN based on long short-term memory (LSTM) learns textual data *t* and a multilayer perceptron deep neural network learns metadata *(W,J,M)*. The cleaned text, represented as an integer-encoded vector, is converted into pretrained tweet word embeddings using GloVe [32] (containing 2 billion tweets, 27 billion tokens, and 1.2 million vocabularies) in the embedding layer. The output of this layer is passed through an LSTM layer for sequence modeling, followed by 1 dropout layer to avoid overfitting and 2 dense ReLU (Rectified Linear Unit) layers. Simultaneously, the metadata of each tweet is passed through 3 fully connected layers with ReLU activation. The outputs of these networks are concatenated into a dense layer, followed by 2 fully connected dense layers, terminating at an output layer with softmax activation, cross-entropy loss, and the adam optimizer. A high-level presentation of this architecture is shown in Figure 2.

To counter the bias caused by class imbalance, for both multiclass and binary classification tasks, we used the class-weight approach to incorporate the weight of each class into the cost function by assigning higher weights to minority classes and lower weights to the majority classes. We also used the SMOTE (Synthetic Minority Oversampling Technique-Nominal Continuous) [33] approach to oversample the minority classes by creating synthetic samples based on their feature space. However, we did not notice much difference between using and not using the synthetic minority oversampling technique. Thus, our final models were trained using the class-weight approach. The hyperparameters for each method were determined using a nested 10-fold cross-validation Bayesian optimization [34].

As the main goal of both supervised and unsupervised label inference models was to minimize the number of false-negative and false-positive inferences, to evaluate the models developed in this study, we used precision, recall, F1, and precision-recall area under the curve (AUC_PR_) metrics.

All the computations and predictive models were implemented using Python 3.7 with TensorFlow 2.0 [35], Keras [36], and Scikit-learn [37] libraries. To facilitate the replication of our study, the code repository of this study is publicly available on GitHub [38].

## Results

### Raw Labels From AMT Workers

**Figure 3:**
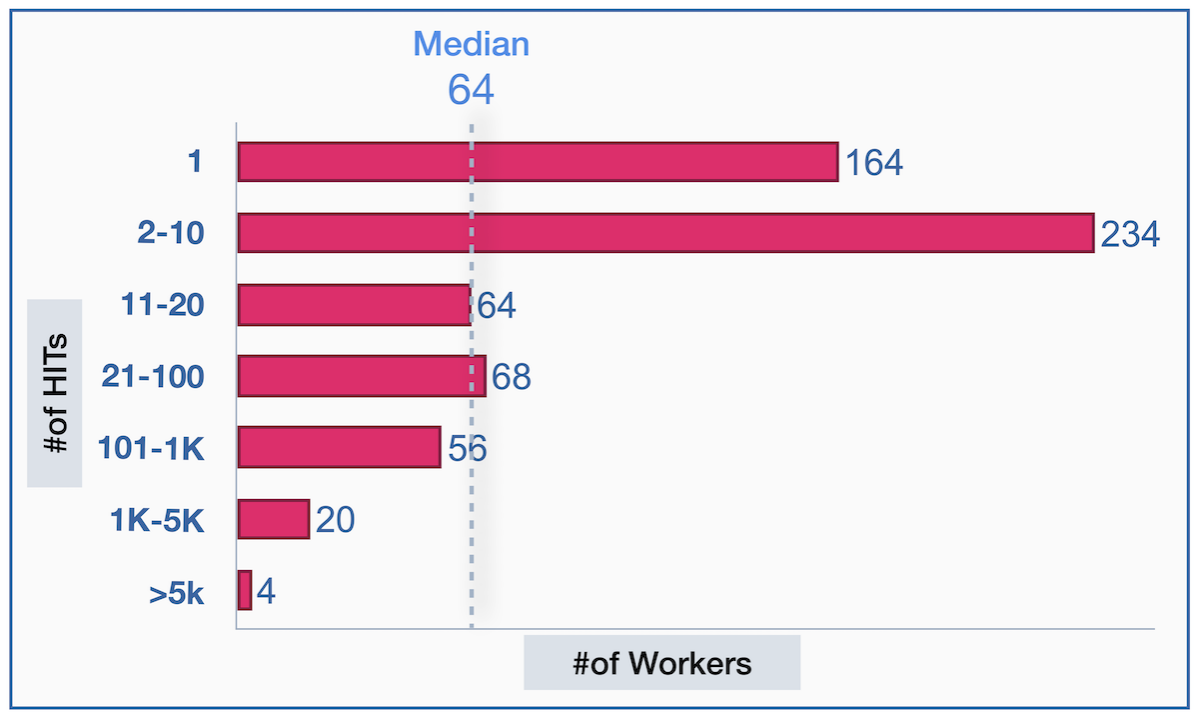
The number of workers who completed different numbers of human intelligence tasks (HITs). Most workers completed a relatively small number of HITs.

In total, 610 unique workers participated in our data labeling tasks and completed 103,911 HITs, from which 5189 HITs were removed as they did not receive 3 valid answers. We approved 98,722 tasks for further analysis. Most workers (530/610, 86.9%) completed <100 HITs, of which 164 completed only 1 HIT. Among the workers who completed >5000 HITs, 1 worker completed 21,801 HITs and 3 workers completed between 5000 and 10,000 HITs (Figure 3). The calculated *LC* for each PASS category for multiclass labeling was 0.54, 0.58, and 0.55 and for binary labeling was 0.75, 0.77, and 0.74 (Table 1). This implies a high level of label inconsistency, prompting the need for further label quality analysis for the development of ML models.

**Table 1:** Details of the collected labels and label consistency (LC) score for each of the physical activity, sleep quality, and sedentary behavior categories. LC ranges from 0 to 1, and the values close to 1 show more consistency among workers’ input.

Type	Tweets, n (%)	LC_multi_	LC_binary_	Workers, n (%)
Physical activity	48,576 (49.2)	0.54	0.75	232 (38)
Sedentary behavior	17,367 (17.6)	0.55	0.74	157 (25.7)
Sleep quality	32,779 (33.2)	0.58	0.77	221 (36.2)
Total	98,722 (100)	0.56	0.75	610 (100)

### Truth Inference

Table 2 describes the ground truth data set of 9000 tweets that was used to train the truth inference models. Table 3 lists the inference results obtained from the 4 unsupervised models and 7 supervised predictive models, including 2 DL models, on the ground truth data set. Each model was evaluated on both binary and multiclass versions of the data set for each PASS category. Among the unsupervised models for physical activity and sleep quality, DS and RY performed better than MV and GLAD for all performance metrics, whereas MV outperformed the other models on the sleep quality data set. Interestingly, for binary inference across all PASS categories, MV outperformed or performed just as well as the other methods, indicating the impact of task complexity on the performance of inference methods.

DL*_meta_* outperforms other methods with the minimum number of false positives (precision: 78%) for the multiclass classification task, but other methods performed better with respect to recall, F1, and AUC_PR_ metrics. Performance on each PASS data set for binary classification did not highlight any individual method constantly performing best. For example, whereas SVM showed the best performance for physical activity, KNN and LR outperformed other models for sleep quality and sedentary behavior, respectively. LR achieved superior performance across all data sets for the multiclass inference task. To analyze this further, we modified the hyperparameters of the LR algorithm presented in Table 3 to stochastic average gradient solver and *l_2_* regularization and the optimizer of the hybrid neural network to stochastic gradient descent and repeated the comparisons. LR still outperformed the neural network model by more than 2% in all metrics. The poor performance of the neural networks in this study could be attributed to the imbalanced ratio of data (per class) to the model parameters (ie, high variance).

**Table 2:** Characteristics of the ground truth data set used to develop and evaluate the supervised and unsupervised inference models.

Variable				Physical activity (n=4000)		Sedentary behavior (n=2000)		Sleep quality (n=3000)
**Labels, n (%)**								
	**Binary**							
		Yes	1629 (40.73)		726 (36.3)		1063 (35.43)	
		No	2371 (59.28)		1274 (63.7)		1937 (64.57)	
	**Multiclass**							
		YY^a^	1629 (40.73)		726 (36.3)		1063 (35.43)	
		YN^b^	550 (13.75)		395 (19.75)		862 (28.73)	
		NY^c^	179 (4.48)		19 (0.95)		52 (1.73)	
		NN^d^	1642 (41.05)		860 (43)		1023 (34.1)	
**Gender, n (%)**								
	Female		1131 (28.28)		576 (28.80)		469 (15.63)	
	Male		1980 (49.50)		906 (45.30)		490 (16.34)	
	Unknown		889 (22.22)		518 (25.90)		2041 (68.03)	
**Age range** **(years), n (%)**								
	≤18		204 (5.10)		170 (8.50)		150 (5)	
	19-29		743 (18.58)		475 (23.75)		331 (11.03)	
	30-39		897 (22.42)		365 (18.25)		249 (8.30)	
	≥40		1267 (31.68)		472 (23.60)		229 (7.64)	
	Unknown		889 (22.22)		518 (25.90)		2041 (68.03)	
**Day of week, n (%)**								
	Sunday		664 (16.60)		325 (16.25)		440 (14.66)	
	Monday		595 (14.88)		307 (15.35)		440 (14.66)	
	Tuesday		493 (12.32)		245 (12.25)		435 (14.50)	
	Wednesday		504 (12.60)		278 (13.9)		)393 (13.10)	
	Thursday		525 (13.12)		270 (13.50)		416 (13.86)	
	Friday		531 (13.28)		274 (13.70)		421 (14.03)	
	Saturday		668 (16.70)		283 (14.15)		2433 (14.43)	
	Unknown		20 (0.50)		18 (0.90)		22 (0.76)	
Time (24 hours), Q1-Q3				10-19		10-19		5-18
Month (range)				February to July		April to September		January to August
**Source, n (%)**								
	Organization		563 (14.08)		179 (8.95)		97 (3.23)	
	Users		3437 (85.93)		1821 (91.05)		2903 (96.77)	

^a^YY: self-reported and recent physical activity, sedentary behavior, and sleep quality experience.

^b^YN: self-reported but not recent physical activity, sedentary behavior, and sleep quality experience.

^c^NY: not self-reported but recent physical activity, sedentary behavior, and sleep quality experience.

^d^NN: neither self-reported nor recent physical activity, sedentary behavior, and sleep quality experience.

**Table 3:** Performance of the truth interference methods using a ground truth data set of 9000 labeled tweets: 4000 physical activity, 2000 sedentary behavior, and 3000 sleep quality tweets. The top 4 rows of each PASS (physical activity, sedentary behavior, and sleep quality) category represent the results of the applied unsupervised truth inference models.

Tweets and method		Precision (%)			Recall (%)			F1 (%)			AUC_PR_^a^ (%)	
		Multiclass	Binary	Multiclass		Binary	Multiclass		Binary	Multiclass		Binary
**Physical activity**												
	MV^b^	72	85	70		*85* ^c^	71		84	56		85
	DS^d^	74	85	68		*85*	70		84	54		85
	GLAD^e^	73	84	70		84	71		83	57		84
	RY^f^	74	85	68		*85*	70		84	54		84
	LR^g^	74	85	*75*		*85*	*74*		*85*	*61*		87
	KNN^h^	74	85	74		*85*	73		84	60		*88*
	SVM^i^	72	*86*	73		*85*	73		*85*	*61*		*88*
	RF^j^	73	85	74		84	73		*85*	60		87
	XGBoost	72	81	72		81	71		81	58		83
	DL_meta_^k^	*79*	84	68		84	73		84	60		78
	DL_text_and_meta_	78	84	70		84	73		84	60		78
**Sedentary behavior**												
	MV	71	82	68		82	68		82	54		80
	DS	70	81	62		81	65		81	48		79
	GLAD	71	79	68		79	68		79	54		77
	RY	70	81	62		81	65		81	48		79
	LR	72	*83*	*72*		*83*	70		*83*	*58*		*81*
	KNN	71	82	71		82	67		82	56		80
	SVM	73	*83*	*72*		*83*	70		*83*	*58*		*81*
	RF	72	*83*	*72*		82	69		*83*	57		*81*
	XGBoost	68	82	69		82	67		82	54		80
	DL_meta_	*78*	80	65		80	*71*		80	56		73
	DL_text/meta_	*78*	80	65		80	*71*		80	56		75
**Sleep quality**												
	MV	78	*89*	74		*89*	75		*89*	61		87
	DS	80	*89*	74		*89*	*77*		*89*	62		87
	GLAD	79	85	75		85	76		85	62		82
	RY	80	*89*	74		*89*	76		*89*	62		87
	LR	76	88	*77*		87	*77*		88	64		88
	KNN	76	*89*	*77*		*89*	*77*		*89*	63		*89*
	SVM	76	88	*77*		88	*77*		88	64		88
	RF	75	*89*	76		*89*	76		*89*	63		*89*
	XGBoost	72	87	72		*89*	72		87	58		87
	DL_meta_	*82*	86	72		86	76		86	63		81
	DL_text/meta_	80	87	72		87	76		87	*65*		82

^a^AUC_PR_: precision-recall area under the curve.

^b^MV: majority voting.

^c^Italicization indicates best performance for the metric and each PASS (physical activity, sedentary behavior, and sleep quality) category.

^d^DS: David and Skene.

^e^GLAD: generative model of labels, abilities, and difficulties.

^f^RY: Raykar algorithm.

^g^LR: logistic regression.

^h^KNN: K-nearest neighbors.

^i^SVM: support vector machine.

^j^RF: random forest.

^k^DL: deep learning.

Across all data sets, supervised models consistently performed better than unsupervised methods. This highlights the value of the context-sensitive information that was used as meta-information when training supervised models. However, on sleep quality, a data set with the same features and level of complexity as physical activity and sedentary behavior data sets, MV appears sufficient for the binary inference task, with supervised models providing little or no improvement.

The hybrid CNN architecture did not provide any gain on either the unsupervised inference models or the supervised predictive models (ie, LR, KNN, SVM, RF, XGBoost, and DL_meta_), and in some ways, underperformed them. It is possible that the LSTM stream could not capture the underlying dynamics of the features because of the inconsistencies between the poorly labeled tasks and the textual features.

### Active Learning

To further explore the feasibility of correcting mislabeled samples, we used pool-based active learning [39] with uncertainty sampling. Pool-based active learning assumes that only a small set of data is labeled, and a large pool of data still needs to be labeled through an iterative learning process. All samples in the pool are queried based on an informativeness measure, which improves the learner’s discrimination ability [40]. In this study, our learners were modeled to query the most ambivalent and uncertain samples. For example, for the binary label inference task, samples for which *p*(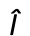 = *l* | *f*) ≈ 0.5 are the most informative samples that may help detect mislabeled samples of the data set through different iterations. We used 5 different base learners with different architectures (ie, RF, LR, KNN, SVM, and XGBoost) with a batch size of 5 and queried the unlabeled pool through 100 iterations.

**Figure 4:**
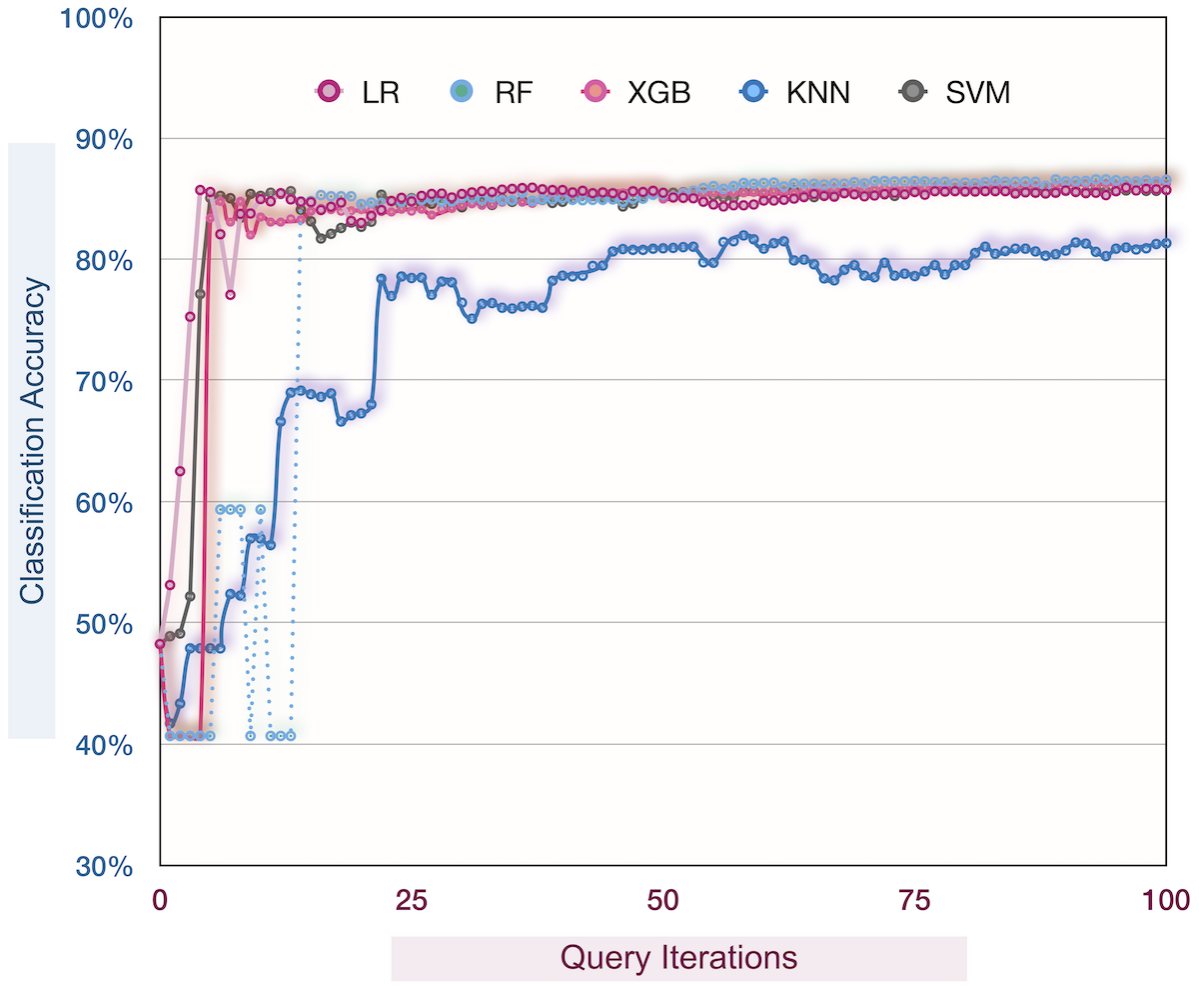
Incremental classification accuracy using pool-based active learning. KNN: K-nearest neighbors; LR: logistic regression; RF: random forest; SVM: support vector machine; XGB: XGBoost.

Our results show that, during the learning process, the accuracy of the classifiers generally increased, slightly degraded at some iterations, and stabilized around iteration 60 for KNN and iteration 20 for other classifiers (Figure 4). Although the active learners in this study could improve their predictive ability through a self-learning process, they failed to correct mislabeled samples and stabilized at performance scores lower than those of the offline learners discussed earlier (Table 3).

## Discussion

### Practical Recommendations

We start this section with some practical recommendations and guidelines on the use of AMT in specific and crowdsourcing in general for developing ML-based public health surveillance systems. Even under the assumption that more advanced artificial intelligence models, including pretrained models on general scope data sets and transfer-learning techniques, can cope with the poor quality of crow-generated labels, the guidelines provided in this study can still improve the implementation, design, and qualification of the crowd-labeling as well as the label inference processes. These guidelines are supported by the results described earlier and the findings and further analysis discussed in the rest of this section.

First, although the demographics of AMT workers are not available, we can still implement the crowdsourcing process in a way that accommodates a greater diversity of workers. A longitudinal labeling process, rather than one-time labeling, allows researchers to monitor the quality of the collected data over time, and mitigates the impact of spammers, irresponsible workers, and workers who are biased or mistake prone. Second, the overall quality of AMT workers can be context-sensitive and vary based on the type of labeling task. For example, the familiarity of the workers in the context of the tasks in the sleep quality data set, contrasting the broad context of physical activity and sedentary behavior concepts, resulted in higher data quality. Researchers should also be aware of the exclusion rate (eg, 5189/103,911, 4.99% in this study) and need to consider this when planning for their study’s budget and design. Third, our study results show that consensus-based inference models that do not consider the task’s features may not always be efficient for integrating crowdsourced labels and thus negatively impact the performance of ML models. Fourth, in addition to qualification requirements to filter crowdsourcing participants, sound and illustrative instruction is a less direct way to increase data quality. During the course of this project, we received nearly 70 emails from AMT workers, with most of them asked about scenarios that were mentioned in the instructions. This implies that the instruction changed their default understanding of the tasks, thereby improving the quality of the labels. Finally, when controlling the quality of workers using a qualification question, we recommend not informing the worker that this technique is being used, as they might guess the questions based on their simplicity.

### Key Findings

#### Information Loss About Label Uncertainty

Despite all the alternative models developed in this study to improve the inference accuracy, there were still considerable discrepancies between workers and the truth labels. These disagreements may be attributable to the underlying uncertainty in the data. Although reducing uncertainty by collecting more labels from more workers might simplify the process of label inference, it limits the learning ability of ML models in modeling the inherent uncertainty of data and prevents them from recovering from the mistakes made early during the inference process [41].

#### Robustness of Inference Models

We observed from our inference results that, regardless of the type of the classification task, none of the 11 methods outperformed other methods across all data sets (Table 3). This indicates that inference methods are sensitive to data set characteristics. For example, the performance of all of the methods on the sleep quality data set is better than that of physical activity and sedentary behavior data sets, indicating the low robustness of these models against the task context.

#### The Importance of Task Features

Compared with supervised models that require a large volume of labeled data to integrate crowd-generated labels, using unsupervised inference models is simple and straightforward. However, this simplicity is gained through the cost of throwing away the contextual characteristics of tasks, which may sacrifice quality in context-sensitive scenarios. For example, the time that a tweet is posted during a day can contribute to the decision about its relevance to physical activity or sleep quality contexts. The importance of these characteristics was far more pronounced in the multiclass inference tasks than in the binary tasks (Table 3), suggesting the need for more complicated models when inferring the truth label of tasks with a high level of uncertainty.

#### The Effectiveness of Qualification Requirements

In this study, we used two levels of quality control: (1) through the task assignment process by accepting only workers with a master qualification and (2) through the design and implementation of the tasks by adding a qualification question to our HITs and iteratively observing workers’ performance based on their answer to this question. Our results show that even though defining these requirements improved the quality of crowd-generated labels to a great extent, 12.45% (498/4000), 13.3% (266/2000), and 7.7% (231/3000) of physical activity, sedentary behavior, and sleep quality tweets, respectively, were still mislabeled by all three workers, regardless of their context or complexity level, indicating the need for further quality assessment of crowdsourced data. These mislabeled samples were not misclassified due to sample uncertainty or difficulty, and our further analysis shows that they were not informative enough (ie, prediction scores) to improve the performance of predictive models through the iterative process of active learning (Figure S4 in Multimedia Appendix 1). Considering the sparsity of the (workers and tasks) matrix in large-scale crowdsourcing tasks, distinguishing irresponsible workers and removing their impact is a challenging task that should be carefully considered when training ML models based on crowd-labeled data. A sample list of low-quality labels for all the PASS categories is provided in Figure S5 in Multimedia Appendix 1.

#### The Impact of Crowd-Generated Labels on the Performance of Predictive Models

To further investigate the reliability of using crowdsourcing for developing ML models, we used bidirectional encoder representations from transformers [42] (ie, bert-base-uncased); a transformer-based model with 12-layer, 768 hidden units, 12 heads; and 110M parameters as a contextual input to our DL model, to classify 4000 physical activity tweets, using our binary truth labels and crowd-generated labels. We used the labels inferred by SVM for the crowd-generated labels, as it outperformed other models on the physical activity data set (Table 3). Interestingly, the model that was trained on our ground truth data set outperformed the crowd-labeled data set on all performance metrics by at least 8% (eg, crowd-labeled: AUC_PR_ of 72%; expert-labeled: AUC_PR_ of 82%). This indicates the importance of the quality of crowd-generated labels in developing ML models designed for decision-making purposes, such as public health surveillance decisions.

### Label Prediction Explanation

**Figure 5:**
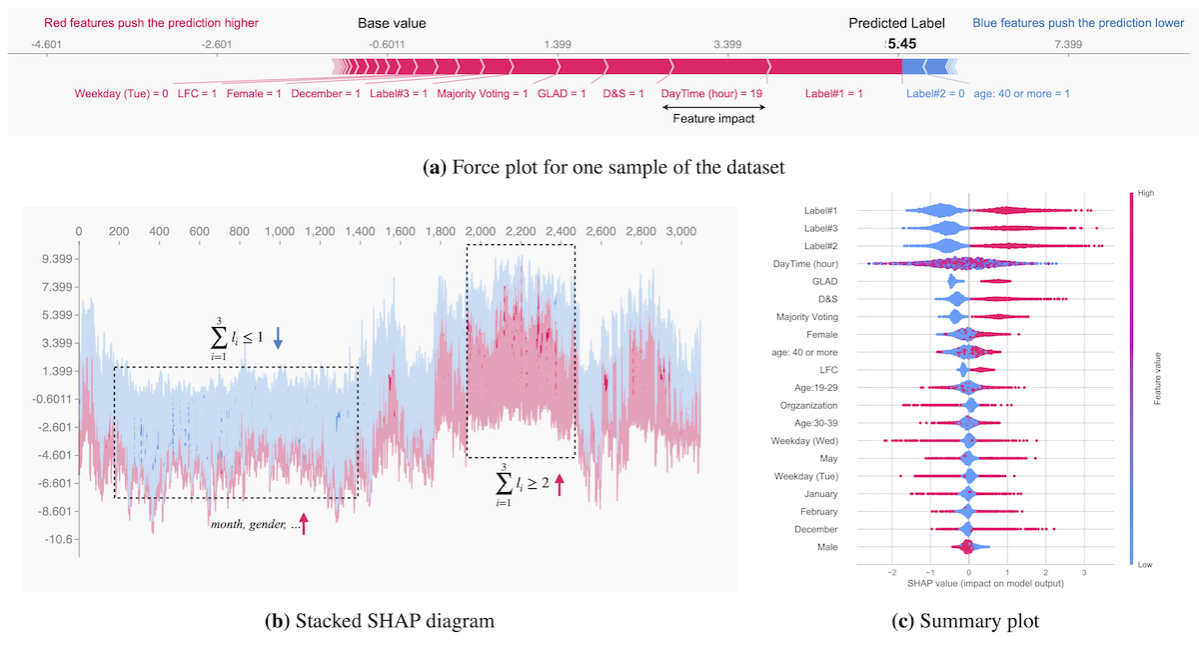
The estimated impact of each piece of meta-information on XGBoost when predicting the truth label. Age is in years. D&S: David and Skene; GLAD: generative model of labels, abilities, and difficulties; LFC: Learning from Crowds (Raykar algorithm); SHAP: Shapley additive explanations.

To interpret the results of our predictive models in terms of the individual contribution of each feature to the prediction results, we used SHAP [23,43]. SHAP calculates the local, instead of global, feature importance for each sample of the data set, which mitigates the risks associated with inconsistency problems in other feature importance techniques. Figure 5A illustrates the interpretation of the prediction using XGBoost on a randomly selected sample of the physical activity data set using SHAP. The red arrows show the features that contribute to the increase, and the blue arrows represent features that contribute to the decrease in the prediction. The width of each arrow indicates the height of its impact. From this example, we can see that *l*_1_=1 and daytime=7pm have the most positive impact on the predicted label, whereas *l*_2_=0 and age ≥40 has the most negative impact.

We further used Shapely values to cluster our data set based on the explanation similarity of samples, using hierarchical agglomerative clustering (Figure 5B). From this figure, we can see that the crowdsourced labels are the most influential features in grouping the samples in our data set. The highlighted areas in this diagram show the samples that have similar force plots, implying the dominant and similar contribution of these features across the physical activity data set.

Using the additive nature of Shapley values, we integrated all the local feature values for each data point and calculated the global contribution (*I*) of each feature. Considering 
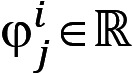
 as the Shapley value of feature *j* for sample *i*, we can calculate the global importance of this feature as 
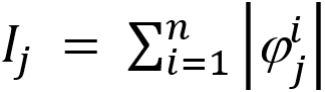
. Figure 5C shows the combination of feature importance (y-axis) and feature effects (colored points) for the most influential features, ordered based on their importance. This plot shows that crowdsourced labels (*l_1_, l_2_,* and *l_3_*), followed by *daytime*, the results of the *inference models*, and *gender* have the greatest impact on the decision-making of XGBoost. From these results, which are extendable to the other predictive models developed in this study, it can be inferred that regardless of the complexity and the architecture of the predictive models, the crowd-generated labels are the factors that most influence predictive models’ prediction. Although meta-information such as *daytime* and *gender* are among the most contributing features (Figure 5C), they still cannot compete with the crowd-generated labels in most of the samples. This can explain the vulnerability of our ML and DL models to the noisy labels of the data set.

To triangulate the dominant impact of the crowdsourced labels, we excluded all the samples for which 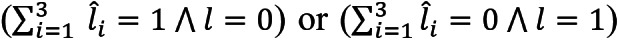 or from our data set for both supervised and unsupervised techniques and achieved an F_1_ score of approximately 99%. This implies that inferring the truth label of crowdsourced data highly depends on the quality of the collected data from the crowd, and even advanced and complex predictive models might not be able to compensate for the poor quality of these data.

### Limitations

This study has several limitations. First, the compensation paid to the workers could impact the quality of the collected labels, and consequently, the evaluation results of this study. Workers may show a higher quality in exchange for higher payments. To investigate this, during the course of the project, we increased HITs’ reward from US $0.03 to US $0.05 and did not notice any significant changes in quality. However, this is still debatable and requires further investigation.

Second, to develop the supervised models, we assumed that all the tasks share the same level of complexity, whereas in reality, some examples are more difficult than others. For example, labeling “I can’t sleep” to a self-reported sleep problem is more straightforward than labeling “I’m kind of envious of anyone who is able to fall asleep before 2am.” We attempted to address this by incorporating inherent task difficulties in the prediction models by developing a hybrid CNN model. However, crowd-generated labels dominated other features of our data set, which had the greatest impact on their inference decisions. Building crowdsourcing models sensitive to the complexity of tasks to allocate more resources (workers) to more difficult tasks is a worthwhile direction for future research.

Third, the way we designed and presented the HITs on AMT could impact the performance of workers in various ways. Considering the central role of people in maximizing the benefits of crowdsourcing services, human factors should be considered when designing crowdsourcing tasks [41]. To address this, we added succinct, precise, and demonstrative instructions to each task and explained each label with an illustrative example (eg, Figure S6 in Multimedia Appendix 1). In addition, through different iterations of data collection, we tweaked the design, presentation, and instructions to ensure that we met the basic usability requirements of task design and presentation.

Fourth, we defined workers’ qualifications based only on their historical performance in completing HITs across AMT (ie, master qualification). Although this provided some degree of quality control on the collected labels, alternative qualification requirements such as workers’ education, work background, and language could have also impacted our study results. To further study the role of qualification filtering, we pilot-tested the labeling process without any qualification requirements for 4500 physical activity tasks. These tasks were completed in <12 hours with a consistency score (*LC*) of <0.5, implying the importance of workers’ quality in developing crowd-labeled intelligent systems.

Fifth, various physical activities, based on their energy requirements in metabolic equivalents (METs), can be categorized into different movement behaviors, such as light (1.6-2.9 METs), moderate (3-5.9 METs), and vigorous (≥6 METs) [44]. However, as the details provided by social media data may not be enough to calculate the MET values, in this study, we only used general terms related to physical activity (eg, physical fitness, exercise, household, sports, or occupational activities) to filter and form the physical activity subset. To ensure that the lists of contextual terms for filtering all the PASS categories are comprehensive enough, in addition to domain-specific ontologies and WordNet [45], we used NLP techniques (eg, topic modeling, language modeling, and lexical analysis) to detect latent word patterns that can be used to identify PASS-related contexts in unstructured text. However, with no impact on the methodology and results of this study, both data collection and population biases (inherent in social media data) should be considered when discussing the data set used for this study.

Despite these limitations, our study is one of the first to rigorously investigate the challenges of using crowdsourcing to develop ML-based public health surveillance systems. Our findings support the argument that crowdsourcing, despite its low cost and short turnaround time, yields noisier data than in-house labeling. On the flip side, crowdsourcing can reduce annotation bias by involving a more diverse set of annotators [41]. This diversity, supported by the diversity of AMT workers [46], is highly beneficial to subjective labeling tasks, such as detecting a sedentary behavior based on a short text, which highly depends on the worker’s understanding of sedentary lifestyles.

The results of this study may inspire future research to investigate and evaluate the application of crowdsourcing for the development of ML-based digital public health surveillance systems deployed and used in national surveillance decision-making. As the potential for success of ML-based digital public health surveillance relies on robust and reliable data sets, a sensitivity analysis of health-related incidents detected by ML-based surveillance models trained on crowd-generated labels versus relevant national datasets is required to ascertain this potential. Moreover, to assess whether our conclusions are sensitive to the background and expertise of participants, further investigation is required using a cohort of experts who are familiar with the public health context under study. Likewise, to untangle the effect of task context and the quality of the crow-generated labels, replicating the approach adopted in this study using other domains, including other public health domains, remains a future work. Finally, as there is a chance that the quality of the crowd-generated labels is subject to the compensation amount, confounded by the socioeconomic characteristics of the participant cohort, future investigations are required to calibrate the results of this study considering these factors.

## Appendix

Multimedia Appendix 1 Additional figures that describe the Amazon Mechanical Turk labeling task, predictive model performance, and incorrectly labeled tweets in more detail.

## Abbreviations

AMT: Amazon Mechanical Turk
AUCPR: precision-recall area under the curve
CNN: convolutional neural network
DL: deep learning
DS: David and Skene
EM: expectation–maximization
GLAD: generative model of labels, abilities, and difficulties
HIT: Human Intelligence Task
KNN: K-nearest neighbors
LR: logistic regression
LSTM: long short-term memory
MET: metabolic equivalent
ML: machine learning
MV: majority voting
NLP: natural language processing
PASS: physical activity, sedentary behavior, and sleep quality
ReLU: Rectified Linear Unit
RY: Raykar algorithm
SHAP: Shapley Additive Explanations
SMOTE: Synthetic Minority Oversampling Technique-Nominal Continuous
SVM: support vector machine

## References

[1] Mavragani A (2020). Infodemiology and infoveillance: scoping review. J Med Internet Res.

[2] Aiello AE, Renson A, Zivich PN (2020). Social media- and internet-based disease surveillance for public health. Annu Rev Public Health.

[3] Sinnenberg L, Buttenheim AM, Padrez K, Mancheno C, Ungar L, Merchant RM (2017). Twitter as a Tool for Health Research: A Systematic Review. Am J Public Health.

[4] Bernardo TM, Rajic A, Young I, Robiadek K, Pham MT, Funk JA (2013). Scoping review on search queries and social media for disease surveillance: a chronology of innovation. J Med Internet Res.

[5] Hossain L, Kam D, Kong F, Wigand RT, Bossomaier T (2016). Social media in Ebola outbreak. Epidemiol Infect.

[6] Lardon J, Abdellaoui R, Bellet F, Asfari H, Souvignet J, Texier N, Jaulent M, Beyens M, Burgun A, Bousquet C (2015). Adverse drug reaction identification and extraction in social media: a scoping review. J Med Internet Res.

[7] Hu H, Phan N, Chun SA, Geller J, Vo H, Ye X, Jin R, Ding K, Kenne D, Dou D (2019). An insight analysis and detection of drug-abuse risk behavior on Twitter with self-taught deep learning. Comput Soc Netw.

[8] Cavallo DN, Tate DF, Ries AV, Brown JD, DeVellis RF, Ammerman AS (2012). A social media-based physical activity intervention: a randomized controlled trial. Am J Prev Med.

[9] Dunn AG, Mandl KD, Coiera E (2018). Social media interventions for precision public health: promises and risks. NPJ Digit Med.

[10] Raghupathi W, Raghupathi V (2014). Big data analytics in healthcare: promise and potential. Health Inf Sci Syst.

[11] Shakeri Hossein Abad Z, Kline A, Sultana M, Noaeen M, Nurmambetova E, Lucini F, Al-Jefri M, Lee J (2021). Digital public health surveillance: a systematic scoping review. NPJ Digit Med.

[12] Paolacci G, Chandler J, Ipeirotis P (2010). Running experiments on Amazon Mechanical Turk. Judgm Dec Mak.

[13] Peer E, Brandimarte L, Samat S, Acquisti A (2017). Beyond the Turk: alternative platforms for crowdsourcing behavioral research. J Experiment Soc Psychol.

[14] Brabham DC, Ribisl KM, Kirchner TR, Bernhardt JM (2014). Crowdsourcing applications for public health. Am J Prev Med.

[15] Kim SJ, Marsch LA, Hancock JT, Das AK (2017). Scaling up research on drug abuse and addiction through social media big data. J Med Internet Res.

[16] Lu W, Guttentag A, Elbel B, Kiszko K, Abrams C, Kirchner TR (2019). Crowdsourcing for food purchase receipt annotation via Amazon Mechanical Turk: a feasibility study. J Med Internet Res.

[17] Ayers JW, Leas EC, Allem J, Benton A, Dredze M, Althouse BM, Cruz TB, Unger JB (2017). Why do people use electronic nicotine delivery systems (electronic cigarettes)? A content analysis of Twitter, 2012-2015. PLoS One.

[18] Yin Z, Fabbri D, Rosenbloom ST, Malin B (2015). A scalable framework to detect personal health mentions on Twitter. J Med Internet Res.

[19] McIver DJ, Hawkins JB, Chunara R, Chatterjee AK, Bhandari A, Fitzgerald TP, Jain SH, Brownstein JS (2015). Characterizing sleep issues using Twitter. J Med Internet Res.

[20] Reece AG, Reagan AJ, Lix KL, Dodds PS, Danforth CM, Langer EJ (2017). Forecasting the onset and course of mental illness with Twitter data. Sci Rep.

[21] Adrover C, Bodnar T, Huang Z, Telenti A, Salathé M (2015). Identifying adverse effects of HIV drug treatment and associated sentiments using Twitter. JMIR Public Health Surveill.

[22] Peer E, Vosgerau J, Acquisti A (2013). Reputation as a sufficient condition for data quality on Amazon Mechanical Turk. Behav Res.

[23] Lundberg S, Lee S (2017). A unified approach to interpreting model predictions - advances in neural information processing systems. Proceedings of the 31st Conference on Neural Information Processing Systems (NIPS 2017).

[24] Wang Z, Hale S, Adelani D, Grabowicz P, Hartman T, Flöck F, Jurgens D (2019). Demographic inference and representative population estimates from multilingual social media data. Proceedings of the World Wide Web Conference.

[25] Shakeri Hossein Abad Z, Butler GP, Thompson W, Lee J Physical activity, sedentary behaviour, and sleep on Twitter: a multicountry and fully labelled dataset for public health surveillance research. JMIR Preprints..

[26] Zheng Y, Li G, Li Y, Shan C, Cheng R (2017). Truth inference in crowdsourcing: is the problem solved?. Proc VLDB Endow.

[27] Sheshadri A, Lease M (2013). Square: a benchmark for research on computing crowd consensus. Proceedings of the First AAAI Conference on Human Computation and Crowdsourcing.

[28] Dawid AP, Skene AM (1979). Maximum likelihood estimation of observer error-rates using the EM algorithm. Appl Stat.

[29] Ipeirotis P, Provost F, Wang J (2010). Quality management on Amazon Mechanical Turk. Proceedings of the ACM SIGKDD Workshop on Human Computation.

[30] Whitehill J, Wu T, Bergsma J, Movellan J, Ruvolo P (2009). Whose vote should count more: optimal integration of labels from labelers of unknown expertise. Proceedings of the 23rd Annual Conference on Neural Information Processing Systems.

[31] Raykar V, Yu S, Zhao L, Valadez G, Florin C, Bogoni L, Moy L (2010). Learning from crowds. J Mach Learn Res.

[32] Pennington J, Socher R, Manning C (2014). Glove: Global vectors for word representation. Proceedings of the 2014 Conference on Empirical Methods in Natural Language Processing (EMNLP).

[33] Chawla NV, Bowyer KW, Hall LO, Kegelmeyer WP (2002). SMOTE: Synthetic Minority Over-sampling Technique. J Artif Intell Res.

[34] Snoek J, Larochelle H, Adams R (2012). Practical bayesian optimization of machine learning algorithms. Proceedings of the Advances in Neural Information Processing Systems 25 (NIPS 2012).

[35] Abadi M, Barham P, Chen J, Chen Z, Davis A, Dean J, Devin M, Ghemawat S, Irving G, Isard M, Kudlur M, Levenberg J, Monga R, Moore S, Murray D, Steiner B, Tucker P, Vasudevan V, Warden P, Wicke M, Yu Y, Zheng X (2016). TensorFlow: a system for large-scale machine learning. Proceedings of the 12th USENIX Conference on Operating Systems Design and Implementation.

[36] Chollet F (2018). Keras: the python deep learning library. Astrophysics Source Code Library.

[37] Pedregosa F, Varoquaux G, Gramfort A, Michel V, Thirion B, Grisel O, Blondel M, Prettenhofer P, Weiss R, Dubourg V, Vanderplas J, Passos A, Cournapeau D, Brucher M, Perrot M, Duchesnay E (2011). Scikit-learn: machine learning in python. J Mach Learn Res.

[38] CrowdSourcing-for-Digital-Public-Health-Surveillance. GitHub.

[39] Lewis D, Gale W (1994). A Sequential Algorithm for Training Text Classifiers.

[40] Laws F, Scheible C, Schütze H (2011). Active learning with Amazon Mechanical Turk. Proceedings of the 2011 Conference on Empirical Methods in Natural Language Processing.

[41] Lease M (2011). On quality control and machine learning in crowdsourcing. Hum Comput.

[42] Devlin J, Chang M, Lee K, Toutanova K (2018). Bert: pre-training of deep bidirectional transformers for language understanding. arXiv.

[43] Lundberg SM, Erion G, Chen H, DeGrave A, Prutkin JM, Nair B, Katz R, Himmelfarb J, Bansal N, Lee S (2020). From local explanations to global understanding with explainable ai for trees. Nat Mach Intell.

[44] Caspersen CJ, Powell KE, Christenson GM (1985). Physical activity, exercise, and physical fitness: definitions and distinctions for health-related research. Public Health Rep.

[45] Miller GA (1995). WordNet: a lexical database for English. Commun ACM.

[46] Difallah D, Filatova E, Ipeirotis P (2018). Demographics and dynamics of mechanical Turk workers. Proceedings of the Eleventh ACM International Conference on Web Search and Data Mining.

