# Supplementary material for: Crowdsourcing for Machine Learning in Public Health Surveillance: Lessons Learned From Amazon Mechanical Turk
Source: J Med Internet Res. 2022 Jan 18;24(1):e28749. doi: 10.2196/28749 (PMC8808350; doi:10.2196/28749)
Supplement: Multimedia Appendix 1 [file jmir_v24i1e28749_app1.pdf]

Multimedia Appendix

Each HIT contained three questions, an illustrative example, and a qualification question. The qualification question was simple enough to detect random answers or automatic bots. Figure S6 represents the instruction posted on top of each HIT related to physical activity which explains the overall context of the question with a detailed example for each choice.

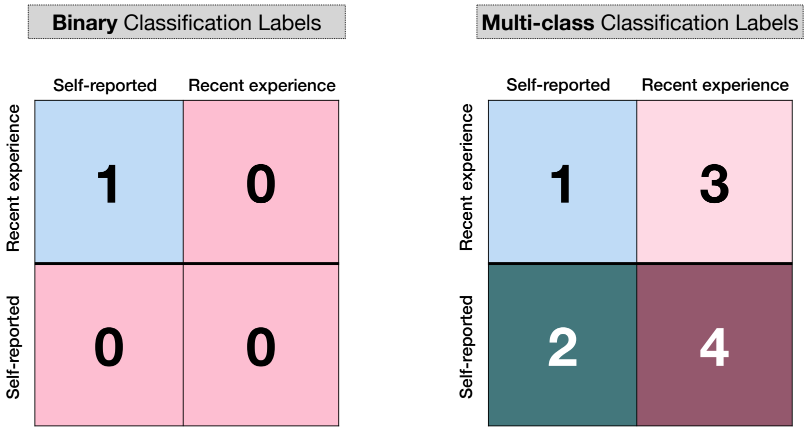

Figure S1. Labels of the binary and multi-class classification tasks.

To further explore the results of using active learning explained in the discussion section, we visualized the prediction score of the mislabeled tasks. As illustrated in figures S4a and S4b, the uncertainty score of these tasks ($\mathcal{U} = 1 - p(\hat{l})$ ranges from 50% to 2%, with the majority of them have $\mathcal{U} < 10\%$.

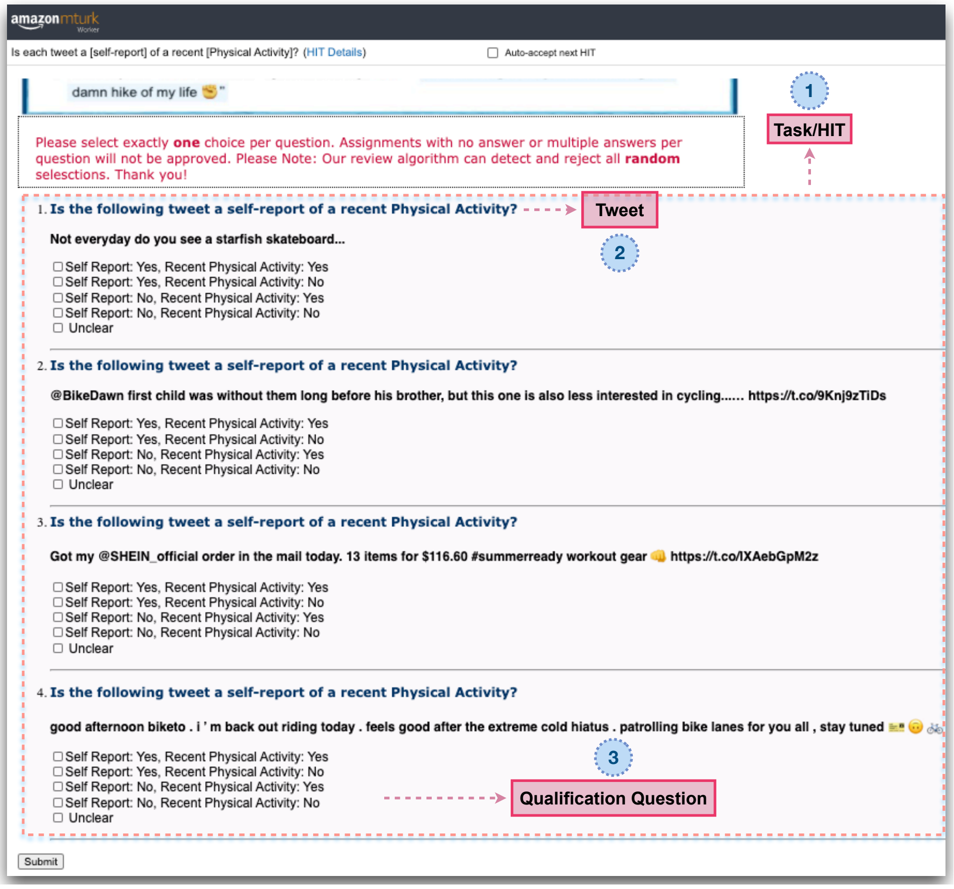

Figure S2. A sample labelling task (i.e., HIT) for the physical activity category, designed for this study.

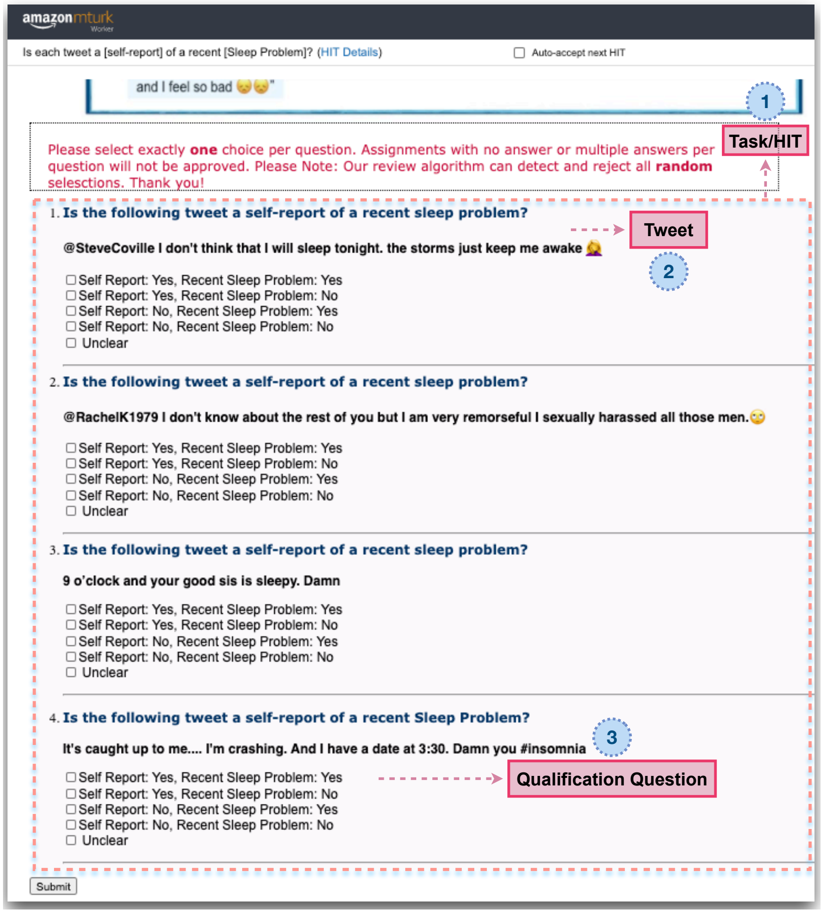

Figure S3. A sample labelling task (i.e., HIT) for the sleep quality category, designed for this study.

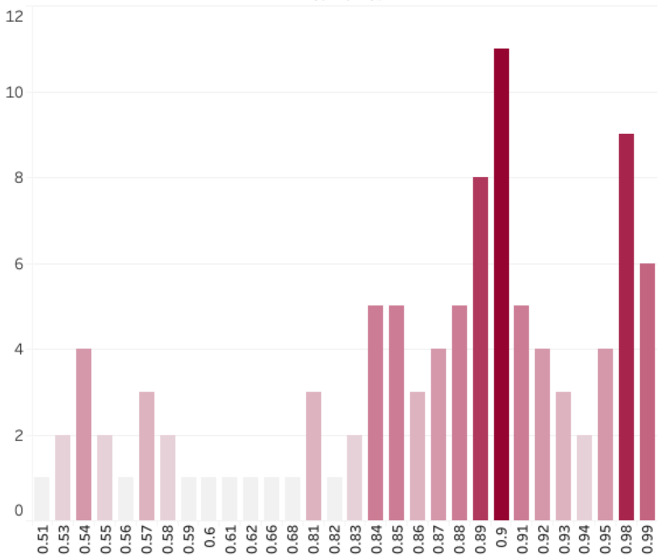

(a) The frequency of LR's scores for the `false positives`

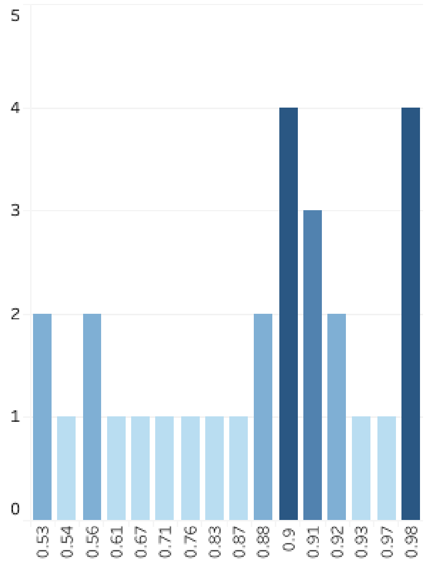

(b) The frequency of LR's scores for the `false negatives`

Figure S4. The frequency of LR's scores for misclassified samples for both `false negatives` and `false positive` samples using the physical activity dataset.

Category	Data/Tweet	W1/W2/W3	Truth	MV	DS	GLAD	RY
PA	So..., last week, I published my post where I reviewed and compared the two home workouts that I have been doing in the past 11 months.	0/0/0	1	0	0	0	0
PA	Two weeks ago our client Doreen couldn't last more than 5 minutes on an exercise bike. Today (after a lot of hard work) she broke 10 minutes on the treadmill and we couldn't be more proud!…	0/1/0	0	0	1	1	1
PA	And so the spring like weather we had for just a day or two is now gone - it's a skating rink outside, freezing rain is so crappy!	1/1/0	0	1	1	1	1
PA	Tuesday.....all the feels💕 #workout #postworkout #community #icantfeelmyarms #familystrong #chipper #smallgymbigheart #yegfitness #fitness #numerouno	0/0/1	1	0	0	1	0
PA	When you finish a class at @BarrysBootcamp on a Saturday... you dance!!	1/0/1	0	1	1	0	1
PA	Great tour of the London Hunt and Country Club this week with the Golf & Club Management students.	1/1/0	0	1	1	1	1
PA	🚨Shoulder and scapula coordination exercise🚨 💪 This is a great follow up to the exercise we posted yesterday	1/0/1	0	1	1	1	1
PA	Con of living alone: how do you do up that tiny clasp at the back of a blouse? My daily gymnastics routine is complete.	0/0/1	1	0	0	0	0
SB	Drove 5 hours for a friend wedding...300 miles	0/0/0	1	0	0	0	0
SB	Finally beat Octopath Traveller took me 130+ hours but i did it. One of my favourite RPGs that i have played ever	1/0/0	1	0	0	0	0
SB	Not looking forward to the 6 hours of bus and subway rides today leaving at 10:45am getting home after midnight probably 😪	0/0/1	1	0	0	0	0
SB	I just scrolled twitter now, I'm 3 hours late but HAPPY BIRTHDAY!!!	1/0/1	0	1	1	1	1
SB	Guys, 6 hours to departure and I couldn't be more excited even though I'm currently sat waiting at my orthopaedic doc's office.	1/1/0	0	1	1	1	1
SB	Time to watch all the complaints come in to #PeelPolice about all the #AmberAlerts today .. do you people have nothing better to complain about .. a child's life is at stake.. Real or Not	1/1/0	0	1	1	0	1
SB	Yeah 48 hrs of straight reading l0l	0/1/0	1	0	0	0	0
SQ	Sorry I got busy then I fell asleep, I need to go back in a few minutes. Yes I do the same thing, but mostly wear one pair the most lol	1/1/1	0	1	1	1	1
SQ	Yeah, complete recovery from last month. I think. I think my sleep schedule is confused getting back to work though. 😅	0/1/0	1	0	0	1	0
SQ	Children have not allowed me to sleep for 5 years... the last 2 years has been a nightmare as my 2yo won't sleep longer than a 3hr stretch on a good night. Some days I feel like I'm losing my mind. What I would give for 8 solid hrs...😴	0/1/0	1	0	0	0	0
SQ	Once this fills up I'll fix my sleep schedule during quarantine	1/0/0	1	0	0	0	0
SQ	Recipe for success: Study while others are sleeping; work while others are loafing; prepare while others are playing; and dream while others are wishing	1/1/0	0	1	1	1	1
SQ	Sleep to get away from ur problems	1/1/0	0	1	1	1	1
SQ	What I experienced is better sleep, relief from PTSD, anxiety, and overall racing thoughts from my accident. I now work with a phycologist out of Calgary,AB and Edmonton helping people through this process	1/1/1	0	1	1	1	1

Figure S5. A sample of low quality labels received from AMT workers for each of the PASS categories (i.e. physical activity (PA), sedentary behaviour (SB), and sleep quality (SQ)). W1/W2/W3 in this figure present the labels received from worker #1, worker #2, and worker #3, respectively. The true label is also compared with the results of unsupervised label inference methods.

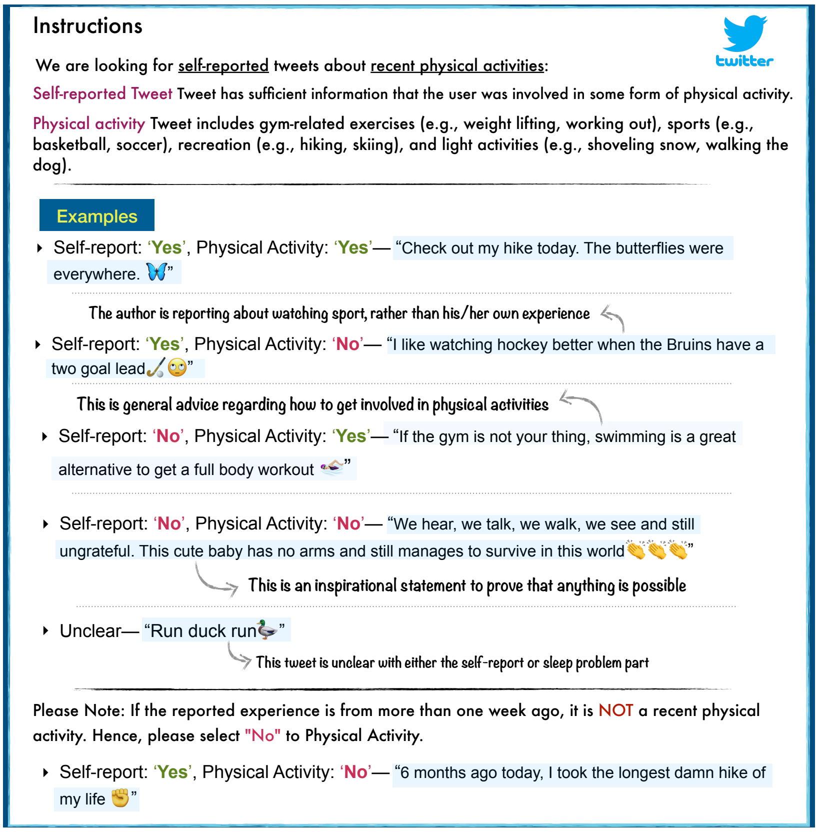
Instructions

We are looking for self-reported tweets about recent physical activities:

Self-reported Tweet Tweet has sufficient information that the user was involved in some form of physical activity.

Physical activity Tweet includes gym-related exercises (e.g., weight lifting, working out), sports (e.g., basketball, soccer), recreation (e.g., hiking, skiing), and light activities (e.g., shoveling snow, walking the dog).

Examples

- Self-report: 'Yes', Physical Activity: 'Yes'— "Check out my hike today. The butterflies were everywhere. 🦋"

The author is reporting about watching sport, rather than his/her own experience

- Self-report: 'Yes', Physical Activity: 'No'— "I like watching hockey better when the Bruins have a two goal lead🏒😳"

This is general advice regarding how to get involved in physical activities

- Self-report: 'No', Physical Activity: 'Yes'— "If the gym is not your thing, swimming is a great alternative to get a full body workout 🏊"

- Self-report: 'No', Physical Activity: 'No'— "We hear, we talk, we walk, we see and still ungrateful. This cute baby has no arms and still manages to survive in this world👏👏👏"

This is an inspirational statement to prove that anything is possible

- Unclear— "Run duck run🦆"

This tweet is unclear with either the self-report or sleep problem part

Please Note: If the reported experience is from more than one week ago, it is NOT a recent physical activity. Hence, please select "No" to Physical Activity.

- Self-report: 'Yes', Physical Activity: 'No'— "6 months ago today, I took the longest damn hike of my life ✊"

Figure S6. The demonstrative instruction posted on AMT for the physical activity task.
